# *In vitro* analysis of material micro-leakage in pit and fissure sealants

**DOI:** 10.6026/973206300200319

**Published:** 2024-04-30

**Authors:** Swati Mittal, Pratyaksha Singh Panwar, Aabha Rawal, Mohomedkaif Mansuri, Anandamoy Bagchi, Monika Pandey, Pratik Surana

**Affiliations:** 1 Department of Pedodontics and Preventive Dentistry, Government Dental College and Hospital, Ahmedabad, Gujarat, India; 2 Department of Dentistry, Government Doon Medical College, Dehradun, Uttarakhand, India; 3 Department of Conservative Dentistry and Endodontics, Faculty of Dental Science, Dharamsinh Desai University, Nadiad, Gujarat, India; 4 Department of Public Health Dentistry, Ahmedabad Dental College and Hospital, Ahmedabad, Gujarat, India; 5 Department of Pedodontics & Preventive Dentistry, Kalinga Institute of Dental Sciences, KIIT Deemed to be University, Bhubaneswar, India; 6 Department of Paediatric and Preventive Dentistry, R.K.D.F. Dental College and Research Center, Bhopal, Madhya Pradesh, India; 7 Department of Pedodontics and Preventive Dentistry, Maitri College of Dentistry and Research Centre, Durg, Chhattisgarh, India

**Keywords:** Pit and fissure sealants, marginal micro-leakage, fuzi vii, clinpro, embrace wetbond

## Abstract

A preferable choice of material offers superior resistance against micro-leakage for clinical applications in preventing dental caries in pits and fissures is of interest. A total of 45 extracted human premolars were cleaned, stored in a saline solution, and randomly divided into three groups, each intended for treatment with one of the sealants: Fuzi VII, ClinPro, and Embrace Wetbond. The application of the sealants followed the manufacturers' instructions strictly. The teeth were subjected to thermal cycling to simulate oral conditions. Marginal micro-leakage was then assessed by dye penetration method using a 0.5% methylene blue dye. Teeth were sectioned, and dye penetration was measured under a stereomicroscope. The results showed that all the tested materials exhibited some degree of micro-leakage. Within the limitations of this *In vitro* study, it was concluded that Embrace Wetbond exhibited superior performance in terms of minimizing marginal micro-leakage among the tested pit and fissure sealants.

## Background:

Dental caries, commonly known as tooth decay, is a prevalent condition characterized by the demineralization of the tooth enamel due to the acidic by-products produced by bacterial fermentation of dietary carbohydrates in the mouth. [1] This process leads to the formation of cavities or holes in the teeth that can cause pain, infection, and in severe cases, loss of the tooth. [2] The morphology and depth of occlusal pits and fissures inherently creates a favourable environment for the accumulation of food particles and bacterial proliferation. This, in turn, limits the effectiveness of mechanical debridement methods in these hard-to-reach areas. [3] As a measure of preventive dentistry, it is advisable to seal these areas prone to caries to safeguard the teeth from decay. The application of occlusal sealants significantly diminishes the risk of caries compared to unsealed teeth and proves to be cost-efficient relative to the use of cements for filling cavities post-decay [4]. Therefore, it is of interest to assess the efficacy of various sealing materials by examining their marginal micro-leakage and the depth of penetration into the occlusal pits and fissures, which are critical factors in determining their protective capability against dental caries.

## Materials and Method:

In the study, a collection of 45 intact maxillary or mandibular premolar teeth, extracted for orthodontic treatments and exhibiting no signs of decay, was analyzed. Following the procurement of these specimens, a thorough cleaning process was undertaken to rid them of any saliva, blood, remaining soft tissue, surface debris, and tartar. This cleaning involved the employment of an ultrasonic scaler to meticulously remove the unwanted materials. As a preparatory step, the teeth underwent a prophylaxis treatment with a mixture of water and pumice, utilizing a prophy cup for application. Subsequently, these samples were rigorously rinsed with water and dried. To ensure they remained hydrated and did not turn brittle, the specimens were stored in a saline solution at room temperature. The study organized the tooth samples into three distinct groups, each comprising 15 teeth. The designations for these groups were based on the type of sealant applied: Group I utilized Fuji VII from GC Corporation in Tokyo, Japan; Group II employed the Clinpro sealant by 3M ESPE, and Group III used Embrace WetBond from Pulpdent in the USA. The procedure for treating the teeth began with the application of a 37% phosphoric acid solution to the occlusal enamel surface for 30 seconds, aimed at etching it. This was followed by a thorough rinse and drying. Sealant was then applied into the grooves according to each manufacturer's instructions. To avoid the formation of air bubbles or voids, a periodontal probe was carefully maneuverer through the fissures. The sealants were cured using a light cure unit on the occlusal surfaces. For Groups II and III, a bonding agent was applied exclusively to the fissures before sealant application. This agent was then polymerized for 20 seconds as per the guidelines provided by the manufacturer.

A layer of adhesive wax was meticulously applied at the apex of each specimen subsequent to the thermo cycling process which involved alternating them between temperatures of 5° and 55°C. This process was repeated for a total of 250 cycles, with each cycle having duration of 10 seconds during which the temperature was carefully maintained. Regular temperature checks were conducted to ensure the accuracy of the thermal conditions. The external surfaces of the specimens were coated with dual layers of nail varnish, maintaining a 2 mm clearance from the boundaries of the sealant. Subsequently, the specimens were submerged in a 5% methylene blue solution for duration of 24 hours. Following immersion, samples were cleansed with water to eliminate any residual dye and then sectioned buccolingually through the sealant employing a high-speed straight handpiece, diamond disk, and water spray. The sectioned specimens were subsequently analyzed using a stereomicroscope at a magnification level of 10, and photographs of the observations were documented.

## Criteria for grading micro-leakage:

The evaluation procedure was implemented employing a 4-point scoring framework by a solitary evaluator, utilizing the Ovrebo and Raadal standards for the assessment of dye infiltration.[5] The Grading Standards for Micro-leakage4 are delineated as follows: Score 0 indicates an absence of dye infiltration, Score 1 is characterized by dye infiltration confined to the external half of the enamel-sealant interface, Score 2 pertains to dye infiltration within the internal half of the enamel-sealant interface, Score 3 is indicative of dye penetration extending into the underlying fissure.

## Results:

The percentage of dye penetration scores obtained with the Chi-squared test is displayed in Table 1. Fuji VII (zero teeth), Clinpro (seven teeth, 46.7%), and Embrace wetbond (twelve teeth, 80%) revealed no dye penetration, according to Ovrebo and Raadal score 0. Fuji VII (three teeth, 20%), Clinpro (three teeth, 20%), and Embrace Wetbond (two teeth, 13.3%), according to Ovrebo and Raadal score I, demonstrated dye infiltration that was limited to the outside half of the enamel-sealant interface. Ovrebo and Raadal score 2; Fuji VII (six teeth, 40%), Clinpro (two teeth, 13.3%), Embrace wetbond (one teeth, 6.67%) showed dye infiltration within the internal half of the enamel-sealant interface. Score 3, Fuji VII (six teeth, 40%), clinpro (three teeth, 20%) and embrace wetbond (Zero teeth) showed dye penetration extending into the underlying fissure. The average micro-leakage score was observed to be the most elevated within the Fuji VII group. Employing the Mann-Whitney test, a statistically significant difference was identified across all three groups concerning the mean micro-leakage score (Table 2).

## Discussion:

Dental caries is now treated as a disease that needs a biological model of care, which represents a significant change in recent years in the way the condition is managed. The model aims to prevent dental cavities by implementing several strategies. Pit and fissure sealing is the most extensively used and highly advised preventive strategy. [6] People at high risk of dental caries, those with numerous carious lesions, and those with deep, narrow pits and fissures in their deciduous and permanent teeth are encouraged to get sealants. [7] Marginal micro-leakage is a paramount concern in the application of pit and fissure sealants, as it can lead to the ingress of bacteria and substances that may promote dental caries underneath the sealant. Therefore, the choice of sealant material is crucial to ensure the longevity and effectiveness of the sealant in caries prevention. This study's findings underscore the importance of material selection, as different sealants exhibited varying degrees of micro-leakage. Data shows that wet bond showed superior performance in minimizing micro-leakage can be attributed to its unique formulation and bonding properties which is in accordance to the study conducted by Joshi *et al.* [8] Unlike traditional sealants, which require a completely dry field for optimal adhesion, Embrace Wetbond is designed to bond in a moist environment, more closely mimicking the intraoral conditions. This characteristic likely contributes to its enhanced sealing ability and reduced micro-leakage. [9] Clin Pro, while not performing as well as Embrace Wetbond, still demonstrated acceptable levels of micro-leakage. Its formulation, which includes fluoride release for added anti-cariogenic benefit, makes it a viable option for sealant applications. The trade-off between its slightly higher micro-leakage compared to Embrace Wetbond and its therapeutic properties may be considered depending on the clinical scenario and caries risk of the patient.[10] Fuzi VII's performance, showing significantly higher micro-leakage, suggests limitations in its formulation or application method that warrants further investigation. It might hint at a less robust bonding mechanism or sensitivity to moisture common in oral environments, which can significantly affect the sealant's effectiveness over time [11]. This study emphasizes that while no material completely eliminates micro-leakage, the choice of sealant can significantly affect the treatment outcome [1]. Clinicians should weigh the benefits and limitations of each material in the context of their patient's specific needs, the clinical setting, and their preference or experience with the application of specific products [2,3]. Furthermore, while this study provides valuable insights, it is conducted *In vitro*, which involves conditions that might not perfectly mimic the complexities of the oral environment. Factors such as masticatory forces, variations in saliva chemistry, and the presence of biofilm were not accounted for, which could affect the real-world performance of these sealants. Therefore, the extrapolation of these results to clinical practice must be undertaken with caution, and there is a clear need for in vivo studies to comprehensively assess the clinical efficacy of these materials over time.

## Conclusion:

A better understanding of pit and fissure sealants' performance, particularly in terms of marginal micro-leakage is described. Embrace Wet bond's superior performance suggests that it is a preferable choice in clinical settings focused on minimizing micro-leakage.

## Figures and Tables

**Table 1:** Distribution of micro-leakage score across three distinct groups

**Groups**	***n***	**Score 0**	**Score 1**	**Score 2**	**Score 3**	**Mean ± SD**
Group I Fuji VII	15	0	3 (20%)	6(40%)	6(40%)	2.45±0.35
Group II ClinPro	15	7(46.7%)	3(20%)	2(13.3%)	3(20%)	1.01±0.55
Embrace Wetbond	15	12(80%)	2(13.33)	1(6.67%)	0	0.35± 0.65

**Table 2:** Intergroup comparison of micro-leakage using Mann-Whitney test

**Groups**	**P Value**	**Interpretation**
I vs. II	0.001	Significant
I vs. III	0.001	Significant
II vs. III	0.001	Significant

## References

[1] Surana P (2024). Bioinformation..

[2] Prabahar T (2022). Int J Clin Pediatr Dent..

[3] Sridhar LP (2016). J Clin Diagn Res..

[4] Mosleh AA (2022). J Indian Soc Pedod Prev Dent..

[5] Ovrebö RC, Raadal M (1990). Scand J Dent Res..

[6] Gooch BF (2009). J Am Dent Assoc..

[7] Naaman R (2017). Dent J (Basel)..

[8] Joshi KH (2023). J Pharm Bioallied Sci..

[9] Prabakar J (2020). Int J Clin Pediatr Dent..

[10] Garg N (2018). J Dent (Shiraz)..

[11] Ganesh M, Tandon S (2006). J Clin Pediatr Dent..

